# Investigation of discriminatory attitude toward people living with HIV in the family context using socio-economic factors and information sources: A nationwide study in Indonesia

**DOI:** 10.7717/peerj.13841

**Published:** 2022-08-03

**Authors:** Nursalam Nursalam, Tintin Sukartini, Heri Kuswanto, Setyowati Setyowati, Devi Mediarti, Rosnani Rosnani, Rifky Octavia Pradipta, Masunatul Ubudiyah, Dluha Mafula, Sirikanok Klankhajhon, Hidayat Arifin

**Affiliations:** 1 Department of Advanced Nursing Care, Faculty of Nursing, Universitas Airlangga, Surabaya, East Java, Indonesia; 2 Department of Statistics, Institut Teknologi Sepuluh Nopember, Surabaya, East Java, Indonesia; 3 Department of Maternity Nursing, Faculty of Nursing, Universitas Indonesia, Depok, West Java, Indonesia; 4 Nursing Major, Politeknik Kesehatan Kemenkes Palembang, Palembang, South Sumatera, Indonesia; 5 Department of Fundamental Nursing Care, Faculty of Nursing, Universitas Airlangga, Surabaya, East Java, Indonesia; 6 Nursing, Universitas Muhammadiyah Lamongan, Lamongan, East Java, Indonesia; 7 Department of Basic and Emergency Nursing, Faculty of Medicine, Public Health and Nursing, Universitas Gadjah Mada, Yogyakarta, Central Java, Indonesia; 8 Faculty of Nursing, Naresuan University, Phitsanulok, Thailand; 9 Department of Medical and Surgical Nursing, Faculty of Nursing, Universitas Padjadjaran, Bandung, West Java, Indonesia

**Keywords:** People living with HIV, HIV, Discrimination, Family, Attitude, Indonesia

## Abstract

**Background:**

The well-being of people living with HIV (PLHIV) remains a concern. In addition to facing discrimination in their communities, many PLHIV have family members who have a discriminatory attitude. This study analyzes the discriminatory attitude toward PLHIV in the family context using socio-economic factors and information sources in Indonesia.

**Methods:**

A cross-sectional study design was adopted using secondary data from the 2017 Indonesian Demographic Health Survey (IDHS). A total sample of 28,879 respondents was selected using two-stage stratified cluster sampling. The study variables are information sources, sex, age, education, residence, earnings, and familial discriminatory attitude. We used the STATA 16.1 software to analyze Chi-square and binary logistics with a 95% confident interval (CI) with a significance of 5% (*p*-value < 0.05).

**Results:**

In Indonesia, familial discriminatory attitude has a prevalence of 72.10%. In the survey, the respondents with access to some information about HIV (AOR: 0.794; 95% CI [0.722–0.873]), women (AOR: 0.768; 95% CI [0.718–0.820]), and those living in rural areas (AOR: 0.880; 95% CI [0.834–0.929]) were the least likely to have a familial discriminatory attitude. Meanwhile, the respondents aged 15–24 years (AOR: 1.329; 95% CI [1.118–1.581]) and those with a secondary level of education (AOR: 1.070; 95% CI [1.004–1.142]) were the most likely to have a familial discriminatory attitude.

**Conclusion.:**

In the study, we found that, the younger the age and the lower the educational level of the respondent, the more likely they were to have a familial discriminatory attitude. The government may consider these factors when designing policies to tackle familial discrimination faced by PLHIV; in particular, education on HIV and AIDS should be promoted.

## Introduction

Discrimination is an act that violates a person’s human rights, and this is no different for people living with HIV (PLHIV) (UNAIDS, 2017). The Joint United Nations Program on HIV and AIDS (UNAIDS) states that PLHIV have a right to the same access to services and facilities as the rest of the population (Headley et al., 2019). Nonetheless, discrimination is still widely practiced, and the belief that PLHIV are dangerous because they transmit HIV and, therefore, must be avoided is prevalent (Halim, Noor & Thamrin, 2020). Discrimination in the community context (Frye et al., 2017; Iliyasu et al., 2017) makes it harder for PLHIV to get a job (Maulsby et al., 2020) and access health and education services (Asghari et al., 2018; Tafuma et al., 2018). As the people closest to PLHIV are often their family (Fitriyani & Waluyo, 2019; Victoryna, Yona & Waluyo, 2019), the family should be a source of support and comfort. However, many families hold discussions, for example, on the shame they feel for having a HIV-positive family member and how they keep this fact a secret. Stigma and discrimination in the family context is present due to a general lack of understanding and access to information (Fauk et al., 2021).

The number of PLHIV in Indonesia in 2018 was 640,000, and the number of deaths caused by AIDS was 38,000. The latter number is an increase of 60% from 2010 (UNAIDS, 2018). PLHIV in Indonesia still experience a fairly high level of discrimination. As many as 40–50% of PLHIV experience discrimination in their communities, and as many as 67–68% experience discrimination from their families and the people closest to them (Fauk et al., 2021; Neuman & Obermeyer, 2013). Based on data from the 2017 Indonesian Demographic Health Survey (IDHS), in the age range of 15–54 years, the attitude that indicates discrimination by the family is 72.1% and 53.95% by young females (Arifin et al., 2022; DHS, 2017).

In an effort to tackle and minimize discrimination against PLHIV, the Indonesian government issued Law No. 21 in 2013 concerning HIV and AIDS prevention (Kemenkes, 2013). In the act, it is stated that the government’s efforts to combat HIV and AIDS include interventions to eliminate discrimination and stigma toward PLHIV and to provide services, especially health services, that are accessible to and optimized for PLHIV (Ibrahim et al., 2022). As part of these efforts, the government has promoted health using mass media to target the wider community and government agencies; this is aimed at empowering PLHIV within their communities and encouraging all community members to not discriminate against PLHIV in health services, education, and employment and in life in general (Kemenkes, 2013; Pranita, 2019; Rakhman, 2019). However, to effectively tackle discrimination, the main target should be the families of PLHIV. Focused interventions to promote education and acceptance among such families should be initiated.

Studies in Indonesia on the factors related to discrimination against PLHIV have been conducted in the general context but not specifically in the family context. Some of the general factors include sexual orientation, gender (Halim, Noor & Thamrin, 2020; Rogers et al., 2018), knowledge (Nursalam et al., 2021), an irrational fear of HIV (Sofia, 2018), religiosity, self-efficacy (Wilandika, 2019), and the bio-psycho-social-spiritual responses of the family (Nursalam et al., 2014). In addition, there are many studies on labeling in social circles (Abbott & Williams, 2015; Woodgate et al., 2017) and the psychological factors related to a discriminatory attitude toward PLHIV (English et al., 2020). In this study, we investigate discriminatory attitude in the family context. Socio-economic demographic factors and information sources, specifically whether Indonesians get their information from the internet, radio, newspaper or magazine, television, health professions, community meetings, seminars or counseling, or school and teachers, have not previously been examined in research in Indonesia. This research is needed to determine the relationship between a discriminatory attitude and the families of PLHIV in Indonesia.

In its description of the discriminatory attitudes encountered by PLHIV in Indonesia, this research aims to provide information about and an understanding of the discriminative attitude among family members of PLHIV. This study adjusts the data based on socio-economic variables and information sources to investigate discriminatory attitude toward PLHIV in the family context in Indonesia.

## Materials and Methods

### Study design

The cross-sectional design of this study was used to discover the determinants of familial discriminatory attitude toward PLHIV in Indonesia. This study used secondary data from the 2017 IDHS (National Population & Family Planning Board, 2018). To use the data set in this study, the researchers obtained permission from the International Inner-City Fund (ICF).

### Setting

This study used secondary data collected in December 2017. The data sets used in this study are the IDIR71FL data set for Indonesian Individuals Recode Phase 7 and the IDMR71FL data set for Indonesian Men Recode Phase 7 (Croft, Marshall & Allen, 2018; DHS, 2017). The data sets were combined to obtain data on both men and women. The population in this study totaled 59,636 respondents. The sample in this study consisted of men and women who had been interviewed, who were aged between 15 and 54 years, and who had heard of HIV and AIDS. The total sample of 28,879 respondents was determined using two-stage stratified cluster sampling. The sample in this study was weighted by the number of provinces in Indonesia with the aim of obtaining the most even distribution of the data possible.

### Variables

The independent variables in this study are information sources, sex, age, education, residence, and earnings. The information sources variable is a combination of several variables that indicate the sources of information about HIV and AIDS accessed by the respondents. These variables are “internet,” “radio,” “newspaper/magazine,” “television,” “health professionals,” “community meeting,” “seminar/counseling,” and “school/teachers.” The variables were re-coded with “yes” and “no” to standardize the information across the different sources. These variables were then combined to form the variable ‘information sources.’ This variable has three categories: “No information” (if “No” was answered on all of the ‘sources of information’ variables), “Some information” (if “yes” was answered on 1–3 variables), and “More information” (if “yes” was answered on 4–7 variables).

The sex variable in this study is a binary, namely men and women (DHS, 2017). The age variable has four categories based on the standard age classification in Indonesia: 15–24 years, 25–34 years old, 35–49 years old, and 50–54 years old (Kemenkes, 2009). The educational variable has four categories: higher education, secondary education, primary education, and no education. This categorization is based on the regulations in Indonesia in Law Number. 20 of 2003 concerning the National Education System in Indonesia (Kemendikbud, 2003). Based on the population consensus in Indonesia, the residence variable is a binary, namely rural and urban (BPS, 2010). The earnings variable has two main categories: “unpaid” (if the respondent does not receive an income) and “paid,” which has three subcategories depending on what form the respondent receives an income in: cash only, cash and in-kind, and in-kind only (DHS, 2017).

The dependent variable in this study is familial discriminatory attitude. This variable is formed from two variables that indicate the presence of a familial discriminatory attitude. These variables are formed as questions: “Would you want the presence of HIV infection in the family to remain a secret?” and “Would you be ashamed if someone in the family had HIV?” (DHS, 2017; Nursalam et al., 2021). The variables were re-coded with “yes” and “no” to standardize the statements, with statements such as “don’t know/not sure/depend” being excluded from the study. The variables were then combined to form the familial discriminatory attitude variable. This variable has two “yes” categories depending on whether the respondent answered “yes” to one or both of the variables and a “no” category if the respondent answered “no” to both variables.

### Data analysis

The data in the study was analyzed using the STATA 16.1 software. Bivariate analysis was conducted using Chi-square, and multivariate analysis was conducted using binary logistics. The adjusted odds ratio (AOR) had a 95% confident interval (CI) with a significance of 5% (*p*-value < 0.05).

### Ethical consideration

The ethical consideration was performed and approved in Indonesia. We registered and requested access to the IDHS dataset and received approval to access and download the same. The 2017 IDHS was approved under the Institutional Review Board (IRB) Findings Form ICF IRB FWA00000845. Written informed consent for each individual was performed by the IDHS. The information about ethical review is available at https://dhsprogram.com/data/Guide-to-DHS-Statistics/Guide_to_DHS_Statistics_DHS-7.htm (DHS, 2017).

## Results

As shown in Table 1, more than 70% of the respondents have a familial discriminatory attitude. More than 80% only access some information about HIV and AIDS. The majority of the respondents were women, with the most common age being between 35 and 45 years. More than 50% of the respondents had attained the secondary education and lived in an urban area. More than 80% earned an income.

**Table 1:** Socioeconomic and demographic characteristics of the respondents (*n* = 28,879).

Characteristics	*n*	%
Familial discriminatory attitude No Yes	8,057 20,822	27.90 72.10
Information resources No information Some information More information	2,685 24,439 1,755	9.30 84.63 6.08
Sex Men Women	7,424 21,455	25.71 74.29
Age 50–54 35–49 25–34 15–24	912 13,825 8,911 5,231	3.16 47.87 30.86 18.11
Education Higher education Secondary education Primary education No education	7,029 16,281 5,435 134	24.34 56.38 18.82 0.46
Residence Urban Rural	17,041 11,838	59.01 40.99
Respondents’ earnings Unpaid Paid	4,081 24,798	14.13 85.87

As shown in Table 2, the results of the bivariate analysis indicate that all of the variables have a significant relationship with familial discriminatory attitude toward PLHIV in Indonesia.

**Table 2:** Bivariate analysis of the determinants of the familial discriminatory attitude found among PLHIV in Indonesia (*n* = 28,879).

Variables	Familial discriminatory attitude				*p*	X^2^
	No		Yes			
	*n*	%	*n*	%		
Information resources No information Some information More information	647 6,890 520	2.24 23.86 1.80	2,038 17,549 1,235	7.06 60.77 4.28	<0.001	22.95
Sex Men Women	1,832 6,225	6.34 21.56	5,592 15,230	19.36 52.74	<0.001	51.58
Age 50–54 35–49 25–34 15–24	238 3,998 2,463 1,358	0.82 13.84 8.53 4.70	674 9,827 6,448 3,873	2.33 34.03 22.33 13.41	<0.001	18.68
Education Higher education Secondary education Primary education No education	2,056 4,457 1,507 37	7.12 15.43 5.22 0.13	4,973 11,824 3,928 97	17.22 40.94 13.60 0.34	0.034	8.68
Residence Urban Rural	4,611 3,446	15.97 11.93	12,430 8,392	43.04 29.06	<0.001	14.61
Respondents’ earnings Unpaid Paid	1,199 6,858	4.15 23.75	2,882 17,940	9.98 62.12	0.023	5.18

**Note:**

*X^2^: chi-square*.

**Figure 1:**
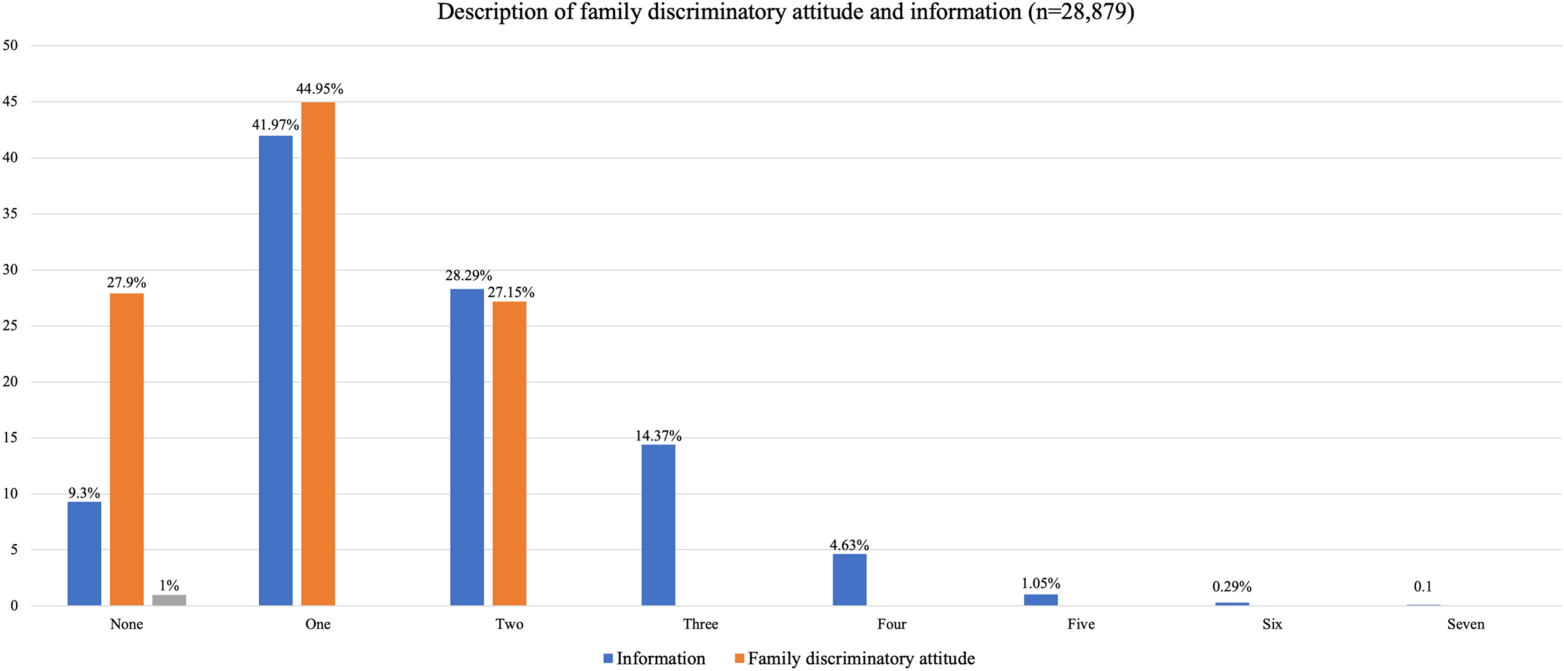
Description of family discriminatory attitude and information.

As shown in Fig. 1, more than 40% of the respondents have a familial discriminatory attitude, while 27.9% do not. A plurality of the respondents (41.57%) access information about HIV and AIDS from one source, while almost 10% have never accessed such information.

As shown in Table 3, the results of the multivariate analysis indicate that the information sources, sex, age, education, and residence variables have a highly significant relationship with familial discriminatory attitude toward PLHIV in Indonesia. The respondents with access to some information about HIV and AIDS were 0.794 times less likely to have a familial discriminatory attitude compared to those with no access to such information (AOR: 0.794, 95% CI [0.722–0.873]). Women were 0.768 times less likely than men to have a familial discriminatory attitude (AOR: 0.768, 95% CI [0.718–0.820]). The respondents aged 15–24 years old were 1.329 times more likely to have a familial discriminatory attitude compared to those who were older (AOR: 1.329, 95% CI [1.118–1.581]). The respondents with a secondary level of education were 1.070 times more likely to have a familial discriminatory attitude compared to those with any other educational level (AOR: 1.070, 95% CI [1.004–1.142]). The respondents living in a rural area were 0.880 times less likely to have a familial discriminatory attitude compared to those living in an urban area (AOR: 0.880, 95% CI [0.834–0.929]). The respondents’ earnings were not related to their attitude toward family members with HIV.

**Table 3:** Multivariate analysis determinants of familial discriminatory attitude among PLHIV in Indonesia (*n* = 28,879).

Variables	Familial discriminatory attitude		
	*p*-value	AOR	95% CI
Information resource No information Some information More information	<0.001 <0.001	1.000 0.794 0.731	[0.722–0.873] [0.635–0.843]
Sex Men Women	<0.001	1.000 0.768	[0.718–0.820]
Age 50–54 35–49 25–34 15–24	0.535 0.073 0.001	1.000 1.052 1.162 1.329	[0.897, 1.234] [0.986, 1.369] [1.118, 1.581]
Education Higher education Secondary education Primary education No education	0.039 0.067 0.747	1.000 1.070 1.083 1.066	[1.004–1.142] [0.994–1.179] [0.724–1.568]
Residence Urban Rural	<0.001	1.000 0.880	[0.834–0.929]
Respondents’ earnings Unpaid Paid	0.359	1.000 1.036	[0.960–1.118]

## Discussion

The results of this study show a significant relationship between the information sources, sex, age, education, and residence variables and familial discriminatory attitude toward PLHIV in Indonesia.

In the study, it was found that people with more access to information are less likely to have a discriminatory attitude toward PLHIV. On average, the respondents had access to 1–3 sources of information about HIV and AIDS. The information packaged in the form of the internet and informative communication media, it can be a form of therapy for PLHIV who are depressed (van Luenen et al., 2018; Zhang et al., 2018). With proper information and understanding, the likelihood of familial discrimination decreases (Namuleme, 2015; Tran et al., 2019). When accepted by their family and an open pattern of information, PLHIV are more likely to have a good quality of life and to be empowered to improve their life (Edianto, Waluyo & Yona, 2019; Fitriyani & Waluyo, 2019; Suardana, Surasta & Erawati, 2020). Good support and information sources, when made easily accessible to communities and families, can facilitate better understanding of HIV, resulting in reduced discriminatory attitude (Li et al., 2006; Xu et al., 2017). With access to information, families are also able to better understand the medical condition of PLHIV, allowing them to provide support and ensure they undergo therapy and continue their personal development.

It was also found that women are less likely to have a familial discriminatory attitude toward PLHIV. This finding is in line with other studies that show men are more likely to discriminate due to emotional instability and misperceptions of HIV (Edianto, Waluyo & Yona, 2019; Ha et al., 2019; Khan, Bilal & Siddiqui, 2019), while women are less likely to discriminate because they have feelings of love and compassion towards PLHIV (Ha et al., 2019; Colombini et al., 2014; Paudel & Baral, 2015). Peer support coupled with information about HIV is likely to reduce the discriminatory attitude of men towards PLHIV.

Furthermore, it was found that young people (15–24 years old) are more likely to have a familial discriminatory attitude toward PLHIV. This finding is in line with other studies that show that younger people are less likely to access the correct information, which may be due to a lack of maturity when receiving information about HIV and AIDS (Emlete et al., 2015; Harper, Lemos & Hosek, 2014). Keeping this in mind, education on HIV should factor in the maturity of the recipient and only gradually become more sophisticated (Andersson et al., 2020; Khan, Bilal & Siddiqui, 2019). In addition, young people stigmatize PLHIV because of the belief that they were infected due to bad behavior such as unsafe sex and drug use and, therefore, should be shunned (Hjerm, Eger & Danell, 2018; WACC, 2014). Families with members who are of a younger age are faced difficulties to process the information properly; young people thus receive the wrong information about HIV and AIDS. Providing correct information is very important because misinformation and misperceptions can increase discrimination. Families, educators, and related parties play an important role in this.

Moreover, it was found that people with a secondary level of education compared to other educational levels are more likely to have a familial discriminatory attitude toward PLHIV. This finding is consistent with research conducted in India, Tasmania, and Bangladesh, which shows that educational level has a highly significant relationship with discriminatory attitude towards PLHIV (Bhagavathula et al., 2015). In this case, level of education is followed by the level of knowledge about HIV and AIDS (Tsai & Venkataramani, 2015). It is concluded that, with a lower level of education, the likelihood of a discriminatory attitude increases due to a lack of information, knowledge, and understanding related to HIV. HIV education should be provided even at a low level of education to reduce the stigma.

Finally, it was found that people living in rural areas are less likely than those in urban areas to have a familial discriminatory attitude toward PLHIV. A number of other studies have confirmed this. The reasons for this rural/urban difference could be a lack of knowledge or exposure to information about HIV in rural areas, so they do not know whether it is dangerous or not (Khan, Bilal & Siddiqui, 2019), beliefs (Chadwick et al., 2018), and a higher tolerance among rural people, making them more likely to accept PLHIV despite the disease (Barrow & Aggleton, 2013). However, this does not mean that rural areas do not need attention. Due to lower education and limited access to information, rural people are more prone to hoax news, which can lead to increased discrimination. Moreover, public awareness and health services are generally lacking in rural areas compared to urban areas (Rhihub, 2020).

### Limitations

This study provides results that are representative of the Indonesian population. The results have been validated using several samples and internationally recognized measurement tools. However, cross-sectional studies have a number of limitations, including the inability to estimate incidence and draw conclusions about causes. Moreover, the information was gathered retroactively and was based on the respondents’ memories. Confounding factors could, therefore, emerge depending on how well the respondents remember their past. Due to data limitations in IDHS, many factors including culture, religion, and belief were not taken into account.

## Conclusions

The results of this study show that having access to information about HIV and AIDS, especially when originating from various sources, makes people less likely to discriminate against PLHIV. In addition, women are less likely than men to discriminate. People of a younger age with a lower educational level, especially when no higher than secondary, are more likely to discriminate compared to people of an older age with a higher educational level. This finding could be related to their level of knowledge and access to information. Finally, families living in rural areas are less likely to discriminate compared to those in urban areas. Based on these research findings, it is recommended that the government consider demographic characteristics to develop better policies to reduce the discriminatory attitude of communities and families toward PLHIV.

## Supplemental Information

10.7717/peerj.13841/supp-1 Supplemental Information 1 Dataset. Raw data exported from the 2017 Indonesian Demographic and Health Survey with the specific variables included in the study for data analysis. Raw data can be used after received an approval from Demographic and Health Survey.

## Additional Information and Declarations

### Data Availability

The following information was supplied regarding data availability: Raw data is available as a Supplemental File.
